# Identity-by-descent mapping for diastolic blood pressure in unrelated Mexican Americans

**DOI:** 10.1186/s12919-016-0041-x

**Published:** 2016-10-18

**Authors:** Xiao-Qing Liu, Jillian Fazio, Pingzhao Hu, Andrew D. Paterson

**Affiliations:** 1 Department of Obstetrics, Gynecology, and Reproductive Sciences, University of Manitoba, Winnipeg, MB R3E 3P4 Canada; 2 Department of Biochemistry and Medical Genetics, University of Manitoba, Winnipeg, MB R3E 3P4 Canada; 3 The Children’s Hospital Research Institute of Manitoba, Winnipeg, MB R3E 3P4 Canada; 4 George and Fay Yee Centre for Healthcare Innovation, University of Manitoba, Winnipeg, MB R3A 1R9 Canada; 5 Program in Genetics and Genome Biology, The Hospital for Sick Children, Toronto, ON M5G 0A4 Canada; 6 Dalla Lana School of Public Health, University of Toronto, Toronto, ON M5G 0A4 Canada

## Abstract

Population-based identity by descent (IBD) mapping is a statistical method for detection of genetic loci that share an ancestral segment among “unrelated” pairs of individuals for a disease. As a complementary method to genome-wide association studies, IBD mapping is robust to allelic heterogeneity and may identify rare inherited variants when combined with sequence data.

Our objective is to identify the causal genes for diastolic blood pressure (DBP). We applied a population-based IBD mapping method to 105 unrelated individuals selected from the family data provided for the Genetic Analysis Workshop 19. Using the genome-wide association study data (ie, the microarray data), chromosome 3 was scanned for IBD sharing segments among all pairs of these individuals. At the chromosomal region with the most significant relationship between IBD sharing and DBP, the whole genome sequence data were examined to identify the risk variants for DBP.

The most significant chromosomal region that was identified to have a relationship between the IBD sharing and DBP was at 3q12.3 (*p* = 0.0016), although it did not achieve the chromosome-wide significance level (*p* = 0.00012). This chromosomal region contains 1 gene, *ZPLD1*, which has been reported to be associated with cerebral cavernous malformations, a disease with enlarged small blood vessels (capillaries) in the brain. Although 24 deleterious variants were identified at this region, no significant association was found between these variants and DBP (*p* = 0.40).

We presented a mapping strategy which combined a population-based IBD mapping method with sequence data analyses. One gene was located at a chromosomal region identified by this method for DBP. However, further study with a large sample size is needed to assess this result.

## Background

As more sequence data become available, one of the challenges is how to identify the disease-causing variants among hundreds of deleterious variants an individual carries [1]. Various research strategies, such as the combined homozygosity mapping and sequence data analysis approach, have been successfully applied [2]. However, other methods are needed for complex diseases and traits that are not autosomal recessive.

Traditional identity-by-descent (IBD) mapping methods have been successfully applied in studies of Mendelian and complex diseases using related cases [3]. With the availability of high-density genetic markers, such as those from genome-wide association and next-generation sequencing studies, it is possible to estimate IBD sharing accurately between 2 randomly chosen individuals in an outbred population for relatively short chromosomal regions, for example, 0.03 to 1 cM instead of 10 to 20 cM from family-based linkage studies [4–7]. In addition, compared to single-marker association studies, IBD mapping methods are more robust to allelic heterogeneity, which has been observed in complex diseases and traits [8].

To date, a few groups have applied IBD mapping methods as a complementary method to genome-wide association studies for complex diseases in large, unrelated samples [5, 9–11]. For each of these studies, the whole genome was scanned in order to compare IBD sharing of each segment in apparently unrelated case-case pairs to that in case-control and control-control pairs. If there were rare causative variants at a locus, the case-case pairs would be expected to share significantly more at this locus than both the case-control and control-control pairs. Several of the studies found genome-wide significant excess IBD sharing for their disease of interest. In contrast, they did not find any chromosomal regions that reached the level of genome-wide significance with genome-wide association methods using the same data.

The population-based IBD mapping methods may also be applied to quantitative traits [12]. In this study, we test the hypothesis that diastolic blood pressure (DBP) levels are related to rare inherited genetic variants that can be identified using the combined IBD mapping and sequence data analysis approach.

## Methods

### Data description

The genome-wide association study (GWAS) data from the families, which had 959 individuals and 472,049 genetic markers, were used for quality control procedures, including relationship and population structure analyses. Unrelated individuals and markers from chromosome 3 of the GWAS data were used for IBD mapping. Chromosome 3 was chosen according to the suggestions from the Genetic Analysis Workshop 19 (GAW19) data contributors.

The DBP from the first time point was used as the outcome. For the individuals with medical treatment for hypertension, 5 mmHg was added to the original DBP values [13]. Multiple linear regression was performed with gender, age, smoking status, and ethnicity (the significant principal components retrieved using the Eigensoft program; see “Quality Control” below) as covariates and the DBP residuals were derived and used to test the relationship between IBD sharing and DBP.

### Quality control

For the GWAS data, individuals were excluded if they had a genotype missing rate of 5 % or greater or a Mendelian error rate of 1 % or greater, and markers were excluded if they were insertion/deletion, had no unique physical location, a missing rate of 5 % or greater, a Mendelian error rate of 1 % or greater, a minor allele frequency (MAF) of 1 % or less, a MAF of 5 % or less with a missing rate of 1 % or greater, or a Hardy-Weinberg equilibrium (HWE) *p* value of less than 0.0001. The markers were further pruned (with *r* ^2^ < 0.33) for relationship and population structure analyses. These quality control procedures were performed using PLINK v1.07 [4].

The PLINK “–genome” results were used to select unrelated individuals. Because Mexican Americans have ancestors with different ethnic backgrounds and because the genetic variants related to DBP may have different allele frequencies in different ethnic groups, the computer program Eigensoft (v4.2) [14] was applied to estimate population structure for the GWAS individuals. The Tracy-Widom statistic was used to evaluate the statistical significance of each principal component. The eigenvectors corresponding to the statistically significant eigenvalues were retrieved and used as covariates in the multiple regression model mentioned under “Data Description” above.

### Identity-by-descent mapping

Beagle Refined IBD (v4.0.r1128) was used to estimate IBD sharing at each location on chromosome 3 [6]. The parameter, *ibdlength* (the minimum IBD length to be detected), was set to 1 cM. Other parameters, *window* (the sliding window that determines the memory usage), *overlap* (overlap between sliding windows), *ibdtrim* (end of a candidate IBD segment trimmed before likelihood calculation), and *ibdwindow* (sliding window for IBD sharing detection), were set to be the number of markers in 12, 1.5, 0.15, and 0.2 cM, respectively. Default settings were used for the remaining parameters. IBD sharing estimation was run 3 times using different seeds. Results from these 3 runs were combined using the ibdmerge.jar utility program (see “Website resources” below).

The physical map was from build GRCh37 of the Genome Reference Consortium 2009. The genetic map was generated by Dr. Brian Browning based on the International Haplotype Map Project (HapMap) Phase II genetic map (see “Website resources” below). If a marker was not in this map, its genetic location was interpolated based on its physical location.

### Test for the relationship between identity-by-descent sharing and diastolic blood pressure

A Perl script was written to create a matrix with the number of unrelated individual pairs × the total number of markers on chromosome 3. The observations in the matrix only had 2 values: 0 if no IBD sharing was observed for pair *i* at marker *j*, and 1 if IBD sharing was observed for pair *i* at marker *j*.

Following the IBD mapping methods proposed for quantitative traits using sibpairs [15–17], we calculated both the squared trait difference (*D*) and squared trait sum (*S*) for the DBP residuals for each unrelated pair. Then, for each marker in the matrix, 2 simple linear regressions were performed:

Regression 1: Y^D^ on the IBD sharing status (π) with estimated slope ${\widehat{\upbeta}}_{\mathrm{D}}$ and variance σ_D_^2^

Regression 2: Y^S^ on π with estimated slope ${\widehat{\upbeta}}_{\mathrm{S}}$ and variance σ_S_^2^

The weighted overall slope estimate is:

$$ \widehat{\beta}=\left(\frac{\sigma_D^2}{\sigma_S^2+{\sigma}_D^2}\right){\widehat{\beta}}_S+\left(\frac{\sigma_S^2}{\sigma_S^2+{\sigma}_D^2}\right){\widehat{\beta}}_D $$

The estimate of the standard error of $\widehat{\beta}$ is:

$$ SE\left(\widehat{\beta}\right)=\sqrt{\frac{1}{\left[\frac{1}{\sigma_D^2}+\frac{1}{\sigma_S^2}\right]\left({\displaystyle {\sum}_{i=1}^n{\widehat{\pi}}_i}\right)}} $$

where ${\widehat{\pi}}_i$ is the observed IBD sharing (0 for no sharing or 1 for sharing) for pair *i*.

Linkage was tested using a one-sided *t* test of the slope estimate. Under the null hypothesis of no linkage, the slope was zero whereas under the alternative hypothesis, the locus was linked to the trait and the slope was negative. The *t* statistic was calculated as the weighted overall slope estimate divided by its standard error:

$$ t=\frac{\widehat{\beta}}{SE\left(\widehat{\beta}\right)} $$

Because the pairs of individuals were not independent from each other, the significance threshold for the real data was obtained using a permutation procedure.

### Whole genome sequence data

For the chromosomal region with the most significant relationship between IBD sharing and DBP, the sequence data were examined to identify the risk variants for DBP. First, variants with no variation (MAF = 0), more than 2 alleles, or a missing rate of more than 15 % were excluded. The remaining genetic variants were annotated with the Combined Annotation-Dependent Depletion (CADD) scaled scores [18]. As recommended by the authors, a genetic variant with a scaled score higher than 20 was regarded as deleterious. The relationships between the deleterious variants and DBP were analyzed using the optimal sequence kernel association test (SKAT-O [19]) with gender, age, smoking status, and the first 3 principal components as covariates.

## Results

After the quality control procedures, 914 individuals and 374,179 markers were selected for relationship and population structure analyses; and 105 unrelated Mexican Americans and 52,216 markers from chromosome 3 of the GWAS data were selected for IBD mapping (Table 1). Three principal components were determined to be significant for population structure and were used as covariates in the multiple linear regression model for DBP. None of the covariates was significantly associated with DBP (ie, with *p* < 0.05); however, the first principal component had a *p* value of 0.1 (ie, the DBP increased 18.2 mmHg for each unit increase in the first principal component).

**Table 1:** Characteristics of the 105 unrelated individuals

Variable	Category	Frequency (%) or Mean (SD, range)
Gender	Male	43 (41 %)
Treatment	Yes	22 (21 %)
Smoking status	Yes	25 (24 %)
Age (years)		56.6 (15.5, 20.3–91.3)
Original DBP (mm Hg)		71.8 (9.3, 51–101)
Adjusted DBP (mm Hg)		72.9 (10.3, 51–101)

For the 105 unrelated individuals, there were 5460 pairs; 1573 (28.8 %) of them had detectable chromosomal segments shared by IBD. The average length of the IBD sharing segments was 1.47 cM with a range of 1.00 to 6.28 cM. The average chromosome-wide IBD sharing rate was 0.0029 with a standard deviation of 0.0025 and a range of 0 to 0.019. For each region, the rate was calculated with the number of pairs with IBD sharing at this region divided by the total number of pairs (ie, 5460).

**Fig. 1:**
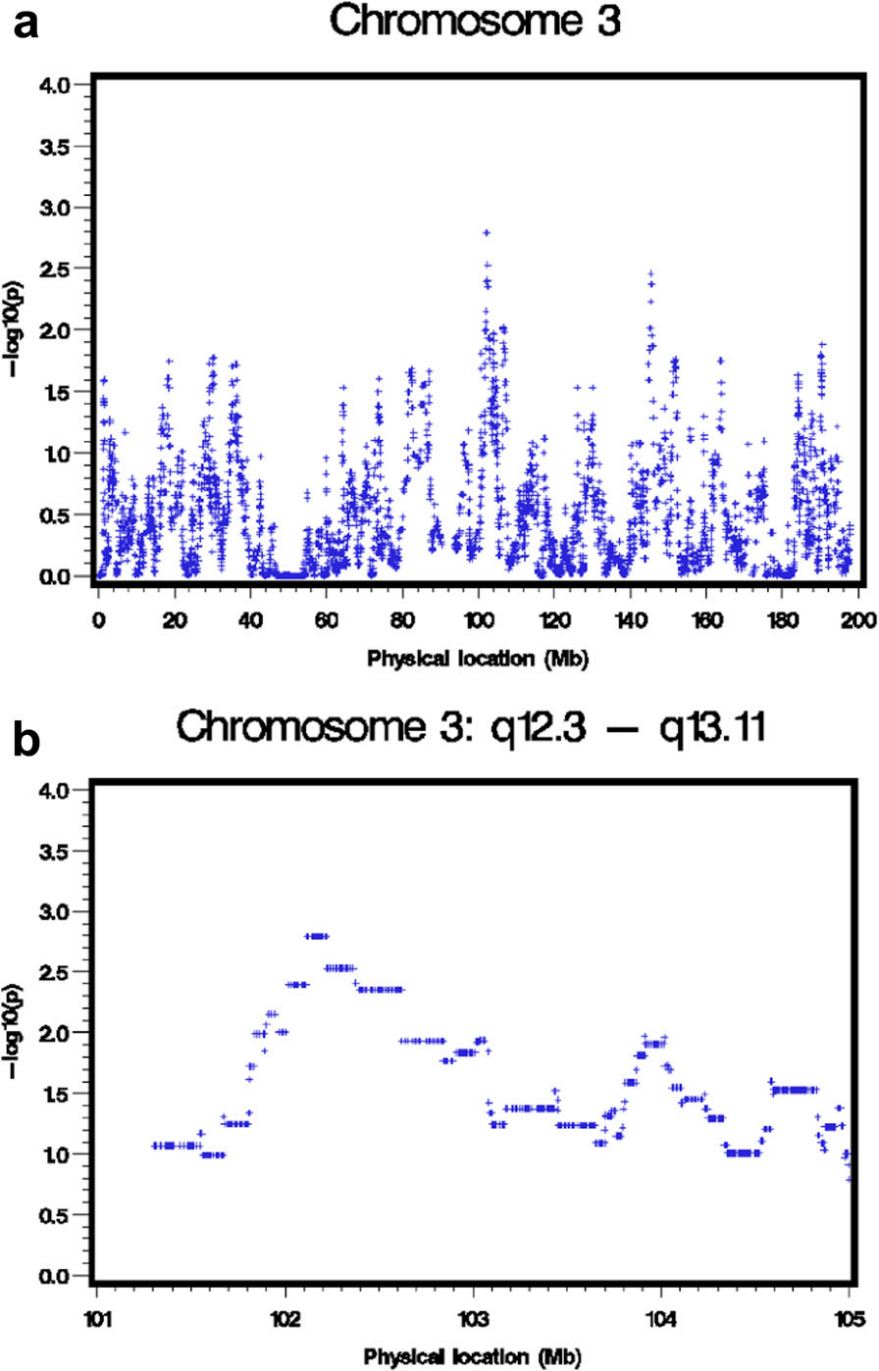
The IBD mapping results for (**a**) chromosome 3 and (**b**) the 3q12.3 to q13.11 region

For the IBD mapping of DBP, the most significant chromosomal region was at 3q12.3 with the peak single-nucleotide polymorphisms (SNPs) located at 101,901,465 to 102,620,049 bp, a region bounded by the GWAS markers that had IBD mapping *p* values of less than 0.01 (Fig. 1). The most significant *p* value for these markers was 0.0016, which did not reach the estimated chromosome-wide significance level 0.00012. For the most significant region, the IBD sharing rate was 0.0057, which was among the top 8 % of the rates for all the regions on chromosome 3.

Of the 105 unrelated individuals, 71 had the whole-genome sequence data. The most significant region overlapped with 1 gene, the zona pellucida–like domain containing 1 gene (*ZPLD1*), which is located at 101,818,088 to 102,196,462 bp. There were 3811 variants at the region after the quality control procedures, 24 of which were identified as deleterious (2 within the gene *ZPLD1*). Based on the result from SKAT-O, these variants were not associated with DBP (*p* = 0.40).

## Discussion

We applied a population-based IBD mapping method to a quantitative trait, DBP. Because of the relatively small sample size (*n* = 105), statistical power to detect the causal chromosomal region was low, and the most significant IBD mapping result did not reach the estimated significance level.

Interestingly, however, the gene at the most significant IBD mapping region, *ZPLD1*, has been reported to be related to cerebral cavernous malformations, a disease with enlarged small blood vessels (capillaries) in the brain, in a patient with a balanced translocation [20]. From Genetic Analysis Workshop 18, Bonner et al. [21] also reported an association between a sparse principal component (which included 28 SNPs at the intergenic region between *ZPLD1* and *MIR548A3*) and systolic blood pressure (SBP) using unrelated individuals from the family data (*n* = 122) and the GWAS genotypes. The Spearman correlation coefficient between the DBP and SBP residuals was 0.59 (*p* <0.0001) in our data. Unfortunately, we were not able to identify causal variants in this gene for DBP. Further study with a large sample size is needed to assess this result.

Using the same IBD sharing estimate method, Browning et al. [6] reported that the average genome-wide IBD sharing rates were 0.015 for a Northern Finland sample and 0.0041 for a United Kingdom (UK) sample [6]. The average chromosome-wide rate was 0.0029 from our Mexican American sample, which was lower than the UK sample, as expected. A higher IBD sharing rate (ie, on average, a larger combined length of detected IBD sharing segments per pair of individuals) can be obtained from an isolated founder population, such as the Northern Finland sample. Current IBD mapping methods are well powered to detect long IBD sharing segments (>1 cM). However, as marker density increases (eg, with whole genome sequencing data), IBD mapping methods will be able to identify IBD sharing status for small segments from outbred populations.

## Conclusions

In summary, we demonstrated a gene mapping strategy for quantitative traits which combines a population-based IBD mapping method with sequence data analyses. However, as a result of the small sample size in this study, more information about this approach and its ability to prioritize causal variants for sequence data analyses should be further explored.
